# Microcirculatory response to experimentally-induced whole body heat stress in healthy humans

**DOI:** 10.1186/cc12152

**Published:** 2013-03-19

**Authors:** A Pranskunas, E Milieskaite, I Maraulaite, L Daniuseviciute, Z Pranskuniene, V Pilvinis, M Brazaitis

**Affiliations:** 1 Lithuanian University of Health Sciences, Kaunas, Lithuania; 2 Lithuanian Sports University, Kaunas, Lithuania

## Introduction

Vasodilation and increased skin blood flow (also sweating) are influential in heat dissipation during heat exposure and exercise. It is unclear how heat stress influences microcirculation. Side dark-field imaging visualizes the blood flow at the capillary level and helps to assess perfusion heterogeneity. Clinical and experimental data show that the sublingual region is clinically relevant for detecting microcirculatory alterations and more represents central microcirculation than cutaneous perfusion. We hypothesize that whole body heat stress may increase capillary density.

## Methods

Eight healthy men with no history of cold and/or heat injury were recruited to this study. Passive body heating was performed by continuous immersion up to the waist in the water bath at 44°C and continued until rectal temperature reached 39.5°C. Before, at the end of whole body heating and 1 hour after heating was ended, systemic hemodynamics and direct *in vivo *observation of the sublingual microcirculation were obtained with sidestream dark-field imaging. Assessment of microcirculatory parameters of convective oxygen transport (microvascular flow index (MFI), proportion of perfused vessels (PPV)), and diffusion distance (perfused vessel density (PVD) and total vessel density (TVD)) was done using a semiquantitative method. Vessels were separated into large (mostly venules) and small (mostly capillaries) using a diameter cutoff value of 20 μm.

## Results

Whole body heating resulted in significantly increased heart rate (*P *= 0.012) and cardiac output (*P *= 0.046) in comparison with baseline variables. One hour after heating was ended, the heart rate remained increased (*P *= 0.012), but cardiac output returned to baseline values. During all observational time the mean arterial pressure remain unaltered. There was no significant difference in MFIand PPV of small vessels at the end of heating and 1 hour after heating in comparison with baseline variables. One hour after heating we observed significant increase in PVD (*P *= 0.046) and TVD (*P *= 0.028) of small vessels regardless of lack of difference at the end of heating (*P *= 0.753 and *P *= 0.075, respectively) in comparison with baseline variables.

## Conclusion

Whole body heating induced time-dependent changes in capillary density.

